# Effect of Genotype and Maternal Affective Disorder on Intronic Methylation of FK506 Binding Protein 5 in Cord Blood DNA

**DOI:** 10.3389/fgene.2018.00648

**Published:** 2018-12-17

**Authors:** Jessica Duis, Olivia H. Cox, Yuelong Ji, Fayaz Seifuddin, Richard S. Lee, Xiaobin Wang

**Affiliations:** ^1^Division of Medical Genetics and Genomic Medicine, Department of Pediatrics, Vanderbilt University Medical Center, Nashville, TN, United States; ^2^Department of Psychiatry and Behavioral Sciences, Mood Disorders Center, Johns Hopkins School of Medicine, Baltimore, MD, United States; ^3^Department of Population, Family and Reproductive Health, Center on the Early Life Origins of Disease, Johns Hopkins University Bloomberg School of Public Health, Baltimore, MD, United States; ^4^Department of Mental Health, Johns Hopkins University Bloomberg School of Public Health, Baltimore, MD, United States

**Keywords:** cord blood, FKBP5, *in utero* environment, DNA methylation, gene-environment interaction, affective disorder, toxic stress

## Abstract

A single nucleotide polymorphism (SNP: rs1360780) in *FKBP5 (FK506 Binding Protein 5)* has been shown to interact with exposure to childhood adversity to promote loss of methylation and increase in gene expression in adults. We asked whether rs1360780 can influence *FKBP5* intronic methylation in the context of exposure to maternal affective disorders *in utero*. Sixty cord blood DNA samples from the Boston Birth Cohort were genotyped at rs1360780 and studied for methylation changes as they relate to genotype and exposure to affective disorders during pregnancy. Linear regression was employed to contrast the risk (TT) genotype to the heterozygous (CT) and homozygous (CC) genotypes with adjustment for potential confounders. The recessive genotype (TT) was associated with increased methylation at multiple CpGs in the *FKBP5* intron 5 region (*p* < 0.01). These findings were enhanced among cases exposed to maternal affective disorders (*p* = 0.02). A human cell line treated with cortisol showed that changes in intron 5 CpG methylation and *FKBP5* expression were inversely associated. These findings suggest that rs1360780 can influence *FKBP5* intronic methylation by acting in *cis* as a methylation quantitative locus and highlight the impact of genotypic risk on methylation *in utero*. Additionally, prenatal stress exposure compounded with the risk genotype may lead to a compensatory increase in methylation.

## Introduction

Stressful environmental conditions can play an important role in neurodevelopment by genetic and epigenetic regulation of glucocorticoid-response genes. Maternal psychosocial variables, when assessed during pregnancy, may be predictive of a child’s neurodevelopmental trajectory and future development of disease. In pediatrics, the term “toxic stress” is often used to describe chronic exposure to adversity with consequential neurobiological outcomes. In the context of fetal development, maternal affective disorders are commonly comorbid with hypothalamic-pituitary-adrenal (HPA) axis dysregulation and hypercortisolemia, and thus categorized as “toxic stress" for the fetus (Kinsella and Monk, 2009; Johnson et al., 2013). Stress regulatory pathways are centered in the hypothalamus and interacts with the pituitary and adrenal glands in response to environmental stimuli. As exemplified in animal models and human cases that show behavioral abnormalities with chronic exposure to stress and elevated glucocorticoids (Starkman et al., 1981; Lee et al., 2011; Fardet et al., 2012; Niwa et al., 2013), a response becomes maladaptive, especially when the stressor persists or occurs during critical stages of development. Maternal hypercortisolemia has been associated with cognitive delays, ADHD (Bergman et al., 2010), and decreased gray matter in the brains of 6–9 year-olds (Buss et al., 2010).

The gene encoding the immunophilin FK506 binding protein 5 (FKBP5) has been implicated in psychiatric, neuroendocrine, and metabolic disorders. A single nucleotide polymorphism (SNP) in *FKBP5*, in particular the T “risk allele” of the C/T SNP rs1360780 located in the second intron, is associated with increased expression of *FKBP5* and dysregulation of the HPA axis. This dysregulation is thought to occur by affecting cortisol recovery following acute exposure to stress (Buchmann et al., 2014) and interfering with glucocorticoid receptor-mediated feedback (Binder et al., 2004). Rs1360780 has been linked to the development of psychiatric disorders with a strong association with stress, such as post-traumatic stress (PTSD) (Binder et al., 2008; Hohne et al., 2014) and major depressive (MDD) disorders (Binder et al., 2004). Recently, rs1370780 has also been associated with intronic methylation in Cushing’s Syndrome, a disease marked by cardiometabolic and psychiatric complications arising from hypercortisolemia (Resmini et al., 2016). In the study, both healthy controls and Cushing’s patients with the TT genotype showed lower intronic methylation, with Cushing’s patients also showing lower methylation at several intronic CpGs compared to controls.

In the presence of glucocorticoids (GCs), the activated glucocorticoid receptor (GR) dissociates from a complex of chaperone proteins that includes FKBP5 and other heat shock proteins, dimerizes with another GC-bound GR, and translocates into the nucleus via dynein-assisted passage (Wochnik et al., 2005). Once in the nucleus, the GR dimer binds to its target DNA, also known as glucocorticoid response elements (GREs), and acts as a transcriptional activator or repressor. One of the target genes is *FKBP5*, and its transcriptional activation by the GR dimer creates a negative feedback loop, whereby hypercortisolemia and glucocorticoid resistance can be attributed to the excess FKBP5 protein that has enhanced affinity for the glucocorticoid receptor (Scammell et al., 2001).

In addition, *Fkbp5* undergoes epigenetic regulation by chronic exposure to glucocorticoids. Studies of mice exposed to high levels of glucocorticoids show reduced methylation of *Fkbp5* at intron 1 in the blood and intron 5 in the brain, most prominently in the hippocampus, hypothalamus, and amygdala (Lee et al., 2010; Sawamura et al., 2016). Additional studies implicate intron 5 as highly susceptible to exposure to glucocorticoids, and show this region to be a GRE that is highly conserved with sequence homology among mammals (Magee et al., 2006). Interestingly, intron 5 is hypomethylated in the adult blood DNA (Klengel et al., 2013), which may indicate this region is developmentally important.

It is thought that effect of the T “risk allele” on increased *FKBP5* expression is moderated by differential methylation at multiple intronic CpGs (Binder et al., 2008; Hohne et al., 2014). For example, exposure to childhood trauma is associated with reduced methylation of intronic regions of *FKBP5*, and this reduction was predominantly observed in the risk allele carriers (Klengel et al., 2013). A possible explanation is that the SNP confers sequence-specific binding of unknown factor(s) that in turn can affect the genomic structure of the region, binding affinities of the intronic GREs to GR, the capacity of CpGs near GREs to undergo glucocorticoid-induced loss of methylation, and the subsequent interaction of the GRs with other transcription factors at the promoter.

In this study, maternal-infant pairs from the Boston Birth Cohort (BBC) were utilized to first examine the association between genotype of *FKBP5* and cord blood methylation patterns to determine the influence of genetic polymorphisms on methylation at birth. We then sought to determine whether “toxic stress” could impact the fetal epigenome *in utero* in much the same way as exposure to adverse events in childhood (Klengel et al., 2013). To this end, “cases” with exposure to maternal affective disorders diagnosed during pregnancy were selected to examine the prenatal gene-environment association between rs1360780 and methylation of intronic CpGs previously implicated in studies of childhood trauma and Cushing’s disease (Klengel et al., 2013; Resmini et al., 2016). Additionally, available maternal samples were utilized for methylation studies to correlate with infant cord blood findings and to examine the molecular evidence of stress exposure on the maternal blood DNA. Finally, we assessed the association between intronic CpG methylation and *FKBP5* expression by examining a glucocorticoid-treated cell line.

## Materials and Methods

### Boston Birth Cohort (BBC)

Participants were recruited at the Boston University Medical Center (BUMC) originally to study adverse birth outcomes, namely preterm birth. In general, the BBC is a multi-ethnic cohort from a range of socioeconomic strata. The study protocol was approved by the Institutional Review Boards of the Boston University Medical Center, the Ann & Robert H. Lurie Children’s Hospital of Chicago (formerly Children’s Memorial Hospital), and the Johns Hopkins University Bloomberg School of Public Health. The methods were carried out in accordance with the approved guidelines. Written informed consent was obtained from mothers. Approximately 6,000 maternal-infant pairs from the BBC (Wang et al., 2002) were queried for maternal diagnosis of affective disorders (anxiety or depression) based upon ICD-9 codes during pregnancy. A total of 343 mothers had a diagnosis of one or more affective disorders. Inclusion required diagnosis at multiple physician prenatal visits. Exclusion criteria included additional confounding diagnoses, medication use for treatment such as selective serotonin reuptake inhibitors (SSRIs), use of drugs or nicotine during pregnancy, unclear diagnosis based on multiple ICD-9 codes used in physician records, and an unavailable cord blood sample for study. The current study used a nested case-control study design, where an initial discovery set was used for identifying potential epigenetic patterns associated with maternal affective disorders, and a second validation set was used for bigger sample size. In both sets, controls were matched to maternal age, parity, socioeconomic status, and ancestry. Preterm birth was initially not excluded in the discovery set but added as a criterion of exclusion in obtaining the full set. The discovery set consisting of two groups, namely 16 cases with exposure to maternal affective disorders and 16 controls, was selected based on maternal age, parity, and ancestry alone (Table 1). Maternal blood samples were obtained within 24–72 h after delivery, and cord blood samples were obtained at delivery. White blood cells were collected in EDTA-treated tubes and subsequently stored in -80°C freezers until genomic DNA extraction. Initially, the discovery set was used to survey 65 promoter and intronic CpGs in *FKBP5*. Since only intron 5 showed significant genotype-specific methylation differences, methylation analysis was limited to CpGs in intron 5 in the validation set.

**Table 1:** Study population characteristics in the initial discovery set (*N* = 32) and the full set (*N* = 60).

		Discovery set (*N* = 32)					Discovery and validation sets (*N* = 60)				
		Controls (*N* = 16)		Cases (*N* = 16)			Controls (*N* = 30)		Cases (*N* = 30)		
		Mean	(*SD*)	Mean	(*SD*)	Pval	Mean	(*SD*)	Mean	(*SD*)	Pval
Gestational age (wk)		39.4	1.3	36.2	4.2	0.008	39.5	1.2	37.8	3.6	0.023
Maternal age (yr)		30.7	7.1	30.8	7.2	0.972	30.0	5.8	29.9	6.2	0.977
Birthweight (g)		3276.8	446.2	2617.9	846.7	0.011	3310.5	405.6	2886.5	722.9	0.007
BMI		27.7	6.3	23.7	5.2	0.056	26.8	5.8	24.8	6.8	0.252
*FKBP5*	CpG-1	16.0	4.7	14.4	4.0	0.315	17.6	4.4	15.9	5.0	0.166
intron 5	CpG-2	10.6	4.1	8.4	2.3	0.065	10.7	3.3	9.4	2.5	0.095
% CpG	CpG-3	11.9	5.1	13.0	7.3	0.607	13.3	5.7	12.5	6.5	0.605
methylation	CpG-4	13.6	4.9	13.7	6.1	0.940	14.0	5.4	12.8	5.6	0.379
	CpG-5	11.9	6.0	13.9	7.1	0.401	12.3	5.8	12.3	6.4	0.998
**Preterm**		**N**	**(%)**	**N**	**(%)**	**Pval**	**N**	**(%)**	**N**	**(%)**	**Pval**
No		16	100%	8	50%	0.002	30	100%	22	73%	0.005
Yes		0	0%	8	50%		0	0%	8	27%	
**Ancestry**		**N**	**(%)**	**N**	**(%)**	**Pval**	**N**	**(%)**	**N**	**(%)**	**Pval**
Black		10	63%	10	63%	1	14	47%	15	50%	1
Caucasian		1	6%	1	6%	1	6	20%	5	17%	1
Hispanic		3	19%	3	19%	1	7	23%	7	23%	1
Haitian		2	13%	2	13%	1	3	10%	3	10%	1


*P-values comparing cases to controls were calculated using a *t*-test for continuous variables and Fisher’s exact test for categorical variables. Preterm delivery is defined as birth prior to 37-week gestation, and values are provided as a percent of total number of samples. % CpG Methylation represents percent DNA methylation determined by bisulfite pyrosequencing. The 5 CpGs assayed are located on Chromosome 6, and their coordinates according to the UCSC HG19 Assembly are: 35,569,922; 35,569,896; 35,569,777; 35,569,757; and 35,569,751.*

### Cell Line

The hypotriploid HEK293 cell line (Atcc.org, Manassas, VA, United States) derived from the human embryonic kidney was cultured using DMEM (ThermoFisher, Waltham, MA, United States) supplemented with 10% fetal bovine serum (Sigma-Aldrich, St. Louis, MO, United States) under standard conditions (5% CO_2_, 37^o^C). Cells were trypsinized and replated in six-well plates before treatment with 1 μM cortisol (CORT; Sigma-Aldrich). Control samples were treated with an equal volume of EtOH as those treated with CORT. Cells were split on the third day of treatment to maintain them in the log phase of growth. After 7 days of treatment, cells were harvested for genomic DNA and mRNA.

### DNA Extraction and Bisulfite Conversion

Genomic DNA (gDNA) from the HEK293 cell line was extracted with the Masterpure DNA Purification Kit, according to the manufacturer’s instructions (Epicentre Biotechnologies, Madison, WI, United States). Concentration of the cell line and human blood gDNA was determined using a Qubit 2.0 Fluorometer (ThermoFisher), and 200 ng of the DNA was used for bisulfite conversion according to the manufacturer’s protocol (EZ DNA Methylation Gold Kit; Zymo Research, Irvine, CA, United States).

### Bisulfite Pyrosequencing

Percent DNA methylation at each of the targeted CpGs was determined by bisulfite pyrosequencing, which measures methylation variation at >90% precision. Briefly, two sets of bisulfite PCR primers were designed to target each of the several intronic regions within the human *FKBP5* gene. PCR reactions were carried out in a Veriti 96-well Thermal Cycler (ThermoFisher) using the ThermoPol Taq DNA Polymerase (New England Biolabs, Ipswich, MA, United States). Two rounds of PCR amplification were performed using 3.5 μL (25 ng DNA) of the bisulfite-converted DNA for the outer PCR and 2 μL of the outer PCR reaction for the nested PCR. One of the nested bisulfite primers was biotinylated and HPLC-purified, allowing it to bind to sepharose beads and become single-stranded, in preparation for bisulfite pyrosequencing. The single-stranded amplicons were annealed to pyrosequencing primers and subjected to primer extension and nucleotide incorporation using the PyroMark Q96 MD pyrosequencer (Qiagen). Pyrosequencing assays were performed once after verification of robust PCR amplification by gel electrophoresis. The pyrosequencer QCpG program determines percent DNA methylation at all of the CpG dinucleotides downstream of the annealed primer. Primers used for each amplicon and pyrosequencing reactions are included in Supplementary Table 1.

### Genotyping

Genotyping of the SNP rs1360780 (C/T) in the second intron of *FKBP5* was performed using the genotyping function on the PyroMark Q96 MD. One round of PCR was performed using 20 ng of gDNA and two PCR primers flanking the SNP, in which one primer was biotinylated and HPLC-purified. In a similar procedure as bisulfite pyrosequencing, this time with non-bisulfite converted DNA, genotypes were determined by primer extension and nucleotide incorporation.

### Gene Expression

Messenger RNA from the HEK293 cell line was extracted using the RNeasy Mini Kit according to the manufacturer’s instructions (Qiagen, Valencia, CA, United States). QuantiTect Reverse Transcription Kit (Qiagen) was used to generate cDNAs for quantitative real-time PCR. Samples without the reverse transcriptase enzyme were used to ensure the absence of contaminating gDNA. All reactions were carried out in triplicate using 1X Taqman master mix (Applied Biosystems, Foster City, CA, United States), 1X Taqman probes for each gene [*FKBP5* and *ACTB* (β-actin)], and 30 ng of cDNA in a total volume of 20 μl. Real-time reactions were performed on an Applied Biosystems 7900HT fast real-time PCR system with standard PCR conditions (50^o^C for 2 min; 95^o^C for 10 min; and 60^o^C for 1 min for 40 cycles). To determine relative expression values, the -ΔΔCt method (Applied Biosystems) was used, where triplicate threshold cycle (Ct) values for each sample were averaged and subtracted from those derived from the housekeeping gene *ACTB*. We used *ACTB*, as previous experiments have shown that its levels do not change with glucocorticoid treatment (Lee et al., 2010; Seifuddin et al., 2017). The Ct difference for a calibrator sample was subtracted from those of the test samples, and the resulting -ΔΔCt values were raised to a power of two to determine normalized relative expression.

### Test for Blood Cell-Type Composition

Five candidate CpGs that characterized each of the five blood cell types on the Illumina 450K platform (CG25939861: CD8+ T-cells; CG27582527: natural killer (NK) cells; CG19276014: B-cells; CG23244761: monocytes; and CG05398700: granulocytes) were assayed by pyrosequencing to test for potential differences in blood cell-type composition between the cases and controls (Houseman et al., 2012; Jaffe and Irizarry, 2014; Lee et al., 2018). Each CpG was hypomethylated in one cell type but hypermethylated in the other four. Using the validation set samples, we observed no significant methylation differences between cases and controls (*P* > 0.34) and among the three genotypes (*P* > 0.62, Supplementary Figures 1A,B). Therefore, methylation values derived from these assays were excluded from analysis.

### Statistical Analyses

All analyses were performed in R software^1^. To assess statistical differences between cases and controls, independent sample *t-*tests were used for continuous variables, and exact Chi-square tests were used for dichotomous variables. Allele and genotype frequencies for the SNP in intron 2 of the *FKBP5* gene were calculated with the gene counting method. We used the HWE exact procedure in the HardyWeinberg R package to carry out tests of Hardy Weinberg Equilibrium in controls and in all participants. We determined that the genotype relationship with CpG methylation appeared to operate according to a recessive mode of inheritance, and thus present all results contrasting the rare homozygous TT genotype versus the heterozygous (CT) or common homozygous (CC) genotype. To formally evaluate the statistical relationship between recessive genotype and methylation, we performed linear regression with CpG value as the dependent variable regressed onto *FKBP5* recessive genotype status, adjusting for maternal age, gestational age, birthweight, BMI, preterm status, stress, and ancestry (African versus else). We also explored the interaction of exposure to a stressful maternal environment and genotype on methylation by incorporating an interaction term between case status and genotype into the regression models. Due to concerns about sample size and power, statistical interaction was only considered for the full sample of 60 individuals and was not carried out in the initial discovery set.

## Results

### Boston Birth Cohort Characteristics and DNA Methylation

The characteristics of the discovery and full sets are listed in Table 1 (See Supplementary Table 2 for individual data). The discovery set consisted of cord blood samples from 16 healthy mothers and 16 case mothers diagnosed with affective disorders (anxiety or depression) during pregnancy. The full set, which includes the discovery and validation sets, consisted of 30 controls and 30 cases. Controls and cases were matched by maternal age, parity, ancestry, and socioeconomic status. In the discovery set, the mean gestational age for controls was 39.4 ± 1.3 (±*SD*) weeks and for cases was 36.2 ± 4.2 weeks (*P* = 0.008). Similar differences in gestational age was observed in the full set (controls: 39.5 ± 1.2 weeks, cases: 37.8 ± 3.6 weeks, *P* = 0.023). These differences were due in part to the significant number of preterm births in the cases in the discovery set (*N* = 8, *P* = 0.002), which remained significant in the full set (*N* = 8, *P* = 0.005), despite the exclusion of additional preterm birth samples in the validation set. Differential gestational age had an obvious effect on birthweight, with the average birthweight for controls at 3,276.8 ± 446.2 g and cases at 2,617.9 ± 846.7 g (*P* = 0.011) for the discovery set and average birthweight for controls at 3,310.5 ± 405.6 g and cases at 2,886.5 ± 722.9 g (*P* = 0.007) for the full set. Therefore, our subsequent epigenetic analyses incorporated gestational age and birthweight as confounding variables.

**FIGURE 1:**
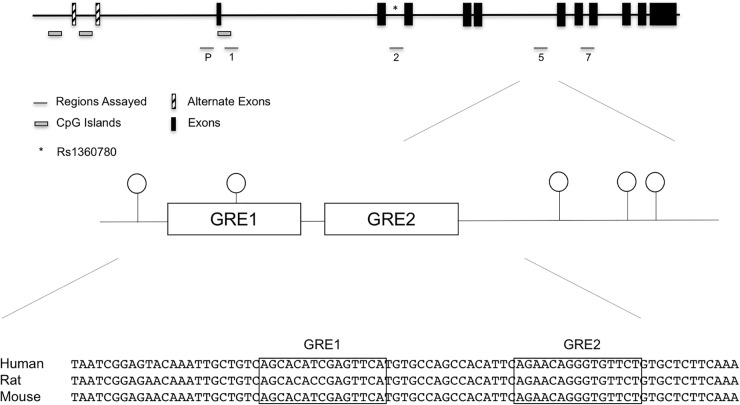
*FKBP5* promoter and intronic CpG positions assayed in the initial discovery set of cases exposed to maternal affective disorders and unexposed controls. Specifically, assays were performed in the promoter, intron 1, intron 2, intron 5, and intron 7. While there were suggestive findings in other regions, statistical significance was unique to the highly conserved intron 5 CpGs. SNP rs1360780 in intron 2 is represented by an asterisk (^∗^). In the bottom panel, sequence alignment of human, rat, and mouse *FKBP5* intron 5 GREs show a high degree of conservation.

We initially used the discovery set to assess the methylation status at 65 promoter and intronic (introns 1,2,5, and 7) CpGs implicated in previous studies (Magee et al., 2006; Lee et al., 2010; Klengel et al., 2013). Genomic locations of the promoter and intronic CpGs that were assayed by bisulfite pyrosequencing are shown in Figure 1, and individual promoter and intronic methylation data for the discovery set are shown in Supplementary Table 3.

Comparing methylation patterns between controls and cases in the discovery set implicated no CpGs of *P-*value-significance (*P* ≤ 0.05). Further, small differences (≤2.3%) in the magnitude of CpG methylation raised concerns regarding their biological significance. For instance, CpG-2 in intron 5 of *FKBP5* trended toward significance (*P* = 0.065) between controls and cases in the discovery set. However, the methylation difference was only 2.2% (Table 1). Similar non-significant results were also obtained for intron 5 CpGs when the discovery and validation sets were examined together (Table 1). These negative findings prompted us to ask whether genotypes could further influence DNA methylation in the discovery set.

### Association Between *FKBP5* SNP rs1360780 and Intron 5 DNA Methylation

All cord blood samples were subsequently genotyped at rs1360780 in intron 2 to assess the potential moderating role of this SNP on DNA methylation. In the discovery set, genotyping showed 14 CC genotypes (reference), 12 CT genotypes (grouped together with the CC homozygotes in subsequent analyses), and 6 TT genotypes (rare homozygous at-risk group). Using the full set, we determined the T allele frequency to be 0.38 and 0.41 in African Americans and Caucasians, respectively. Genotype frequencies were 0.35, 0.50, and 0.15 for the CC, CT and TT genotypes, respectively. We observed no significant deviations from the Hardy Weinberg equilibrium (HWE) in the full sample of 60 individuals (*P-*value for HWE = 1.0) or in the 30 controls (*P* = 1.0).

**FIGURE 2:**
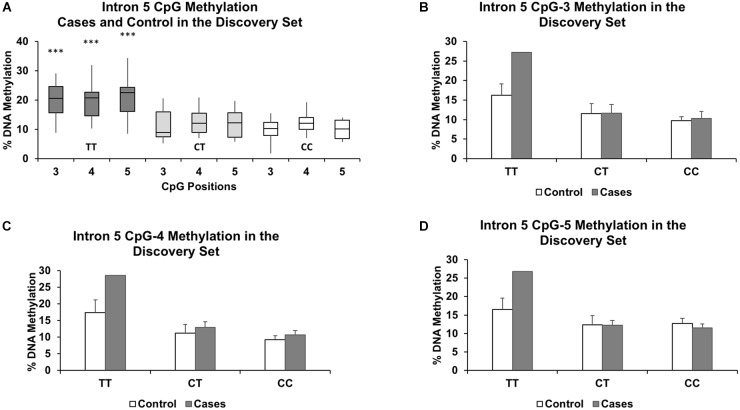
Genotype effect of rs1360780 on DNA methylation in the discovery set at CpG positions 3, 4, and 5 of *FKBP5* intron 5. **(A)** Boxplots show increased DNA methylation in the genotype TT compared to the genotypes CT and CC. ^∗∗∗^ indicates *P-*values < 0.001 in mean methylation difference between TT and CT+CC genotypes for each CpG. Methylation levels for each genotype further segregated by case status for CpG-3 **(B)**, CpG-4 **(C)**, and CpG-5 **(D)** show a pronounced increase in methylation in the TT genotype in the cases compared to controls. The bar graphs are represented as mean ± SEM. The ability to generate *P-*values is impeded by the small sample size of the TT genotype.

We segregated the samples based on genotype, with CC and CT samples grouped together and compared against TT. Our grouping together of the CC and CT genotypes was based on our observation that there were no significant methylation differences between the two genotypes at all intronic CpGs assayed in the discovery set (*P* > 0.57 and methylation differences < 1.4%). Figure 2A shows similar methylation levels between the CC and CT genotypes and their lower methylation levels to those of the TT genotype for three CpGs in intron 5. We also focused our investigation only on the intron 5 CpGs in the validation set and the full set, as we observed no intronic CpGs elsewhere that were significantly different (*P* > 0.05 and %DNA methylation differences less than 5%) between controls and cases and between TT and CC+CT genotypes in the discovery set.

We first performed linear regression analysis in the discovery set, contrasting the recessive TT genotype group with the reference group consisting of the combined CT and CC genotypes, regardless of case-control status. Effect sizes, their corresponding 95% confidence intervals, and *P-*values are displayed in Table 2 for both unadjusted and adjusted models. In the adjusted model, several covariates were used in the analysis: gestational age, maternal age, maternal body mass index (BMI), birthweight, preterm delivery, and ancestry. Three of the five unadjusted CpGs in the discovery set (both controls and cases) showed significant increases in average methylation levels between the two genotype groups prior to adjustment (CpG-3: 9.1%, *P* = 4.9x10^-4^; CpG-4: 7.8%, *P* = 0.001; and CpG-5 10.1%, *P* = 1.9x10^-4^), and the same three CpGs remained significant following adjustment (CpG-3: *P* = 0.002. CpG-4: 0.009; and CpG-5: *P* = 0.003; Table 2).

**Table 2:** Discovery set and full set data comparing genotype at rs1360780 located in intron 2 to % methylation at all 5 CpGs assayed in intron 5 of *FKBP5.*

Intron 5 % CpG methylation					Unadjusted					Adjusted^∗^				
CpGposition	CC and CT	SD	TT	SD	Beta	SE	LCI	UCI	Pval	Beta	LCI	UCI	SE	Pval
**All cases+controls (*N* = 60)**		
CpG-1	16.7	4.9	17.0	4.0	0.30	1.7	–3.2	3.7	0.864	0.12	1.9	–3.6	3.8	0.951
CpG-2	9.7	2.8	11.6	3.5	1.92	1.1	–0.2	4.0	0.076	2.06	1.2	–0.3	4.4	0.080
CpG-3	12.3	5.3	16.3	9.2	3.92	2.2	–0.4	8.2	0.074	4.21	2.3	–0.5	8.9	0.080
CpG-4	12.7	4.5	17.1	8.7	4.41	1.9	0.6	8.2	0.025	4.92	2.1	0.8	9.1	0.021
CpG-5	11.5	4.7	17.1	10.2	5.67	2.1	1.5	9.8	0.008	5.95	2.3	1.4	10.5	0.011
**All cases only (*N* = 30)**		
CpG-1	15.7	5.1	17.8	3.6	2.15	3.0	–4.1	8.4	0.487	2.23	3.3	–4.7	9.1	0.509
CpG-2	9.1	2.4	12.1	2.0	3.06	1.4	0.1	6.0	0.041	3.12	1.6	–0.3	6.5	0.071
CpG-3	11.2	5.2	24.6	4.9	13.46	3.1	7.1	19.9	0.000	14.72	3.5	7.3	22.1	0.001
CpG-4	11.4	3.6	25.1	6.0	13.72	2.3	9.0	18.5	0.000	15.07	2.8	9.3	20.8	0.000
CpG-5	10.9	4.4	25.3	8.0	14.44	2.9	8.5	20.4	0.000	15.72	3.4	8.7	22.8	0.000
**Cases+controls in discovery set (*N* = 32)**		
CpG-1	14.9	4.4	16.7	4.3	1.82	2.0	–2.2	5.8	0.364	0.29	2.2	–4.2	4.8	0.895
CpG-2	8.9	3.2	12.3	3.4	3.42	1.5	0.4	6.4	0.026	3.02	1.7	–0.6	6.6	0.095
CpG-3	10.7	4.6	19.9	7.4	9.12	2.3	4.4	13.9	0.000	10.71	3.0	4.4	17.0	0.002
CpG-4	12.2	3.6	19.9	7.8	7.76	2.1	3.5	12.0	0.001	8.09	2.8	2.2	14.0	0.009
CpG-5	11.0	4.2	21.1	8.9	10.15	2.4	5.3	15.0	0.000	10.74	3.2	4.1	17.4	0.003
**Only cases in discovery set (*N* = 16)**		
CpG-1	14.2	4.2	15.9	1.7	1.64	3.1	–4.9	8.2	0.602	0.63	3.6	–8.2	9.5	0.867
CpG-2	8.0	2.3	11.0	0.0	2.97	1.6	–0.6	6.5	0.093	2.64	2.7	–3.9	9.2	0.361
CpG-3	11.0	5.0	27.2	2.7	16.22	3.7	8.3	24.1	0.001	21.28	5.5	7.9	34.7	0.008
CpG-4	11.8	3.0	26.7	7.4	14.91	2.6	9.2	20.6	0.000	17.11	4.3	6.7	27.6	0.007
CpG-5	11.8	4.0	28.6	8.1	16.79	3.3	9.6	24.0	0.000	19.21	5.3	6.3	32.2	0.011


*Results shown indicate comparison of genotype to methylation regardless of case (prenatal exposure to affective disorders) and control status and then further investigate the association of case and control status with genotype to affect methylation. *P-*values were calculated using linear regression for continuous variables and Fisher’s exact test for categorical variables. ^∗^Adjustments were made for gestational age, maternal age, maternal body mass index (BMI), birthweight, preterm delivery, and ancestry.*

**FIGURE 3:**
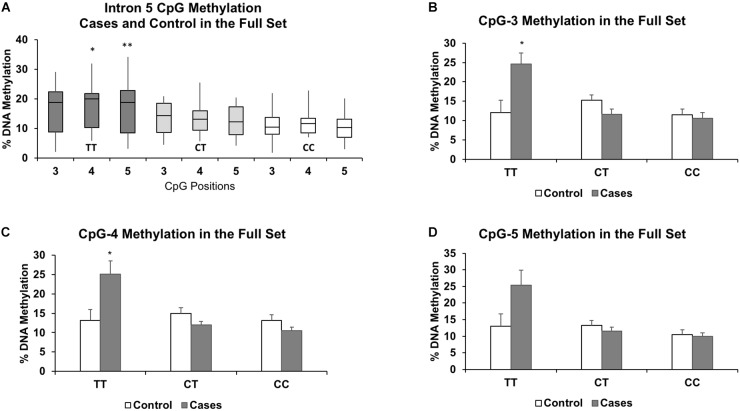
Results of the full set show similar associations between increased methylation and the TT genotype for CpGs 3-5 **(A)**. The results also highlight the similarities in DNA methylation levels between the heterozygous (CT) and protective homozygous (CC) genotypes. Methylation levels for each genotype further segregated by case status for CpG-3 **(B)**, CpG-4 **(C)**, and CpG-5 **(D)** show a pronounced increase in methylation in the TT genotype in the cases compared to controls. The bar graphs are represented as mean ± SEM. For both the boxplots and the bar graphs, ^∗^ and ^∗∗^ indicates *P-*values < 0.05 and < 0.01, respectively, in mean methylation difference between TT and CT+CC genotypes for each CpG.

We then performed another methylation analysis by including additional validation samples to achieve the full set of sixty individuals (both controls and cases). Combining the additional validation samples with the discovery set resulted in similar methylation patterns among the TT, CT, and CC genotypes as in the discovery set alone (Figure 3A). We also re-performed the linear regression, and CpG-4 and CpG-5 still remained significant (adjusted *P* = 0.021 and *P* = 0.011, respectively), albeit with reduced differences in DNA methylation between the two genotype groups (4.4 and 5.7% methylation differences, respectively). (See effect sizes, 95% confidence intervals, and *P-*values provided in Table 2).

### Effect of SNP rs1360780 and *in utero* Exposure to Maternal Toxic Stress on DNA Methylation

Given the significant relationship between the rs1360780 genotype groups and methylation status of several intron 5 CpGs, we then asked whether *in utero* exposure to “toxic stress” could influence the genotype-dependent methylation observed in the cord blood. Therefore, we subsetted our analysis to the cord blood samples of infants who were exposed to maternal affective disorders during gestation. In the discovery set, there were 4 controls and 2 cases with the TT genotype, 5 controls and 7 cases with the CT genotype, and 7 controls and 7 cases with the CC genotype. At intron 5 in the discovery set only, we observed higher methylation levels that were associated with the recessive TT genotype at CpG-3 (+11.0%), CpG-4 (+10.2%), and CpG-5 (+11.2%) in the cases compared to the controls (Figures 2B–D). However, since only two of the cases were of the TT genotype, statistical significance could not be determined. Therefore, we assessed whether the methylation differences were statistically significant between the controls and cases (both were matched by maternal age, parity, ancestry, and socioeconomic status) in the full set.

In the full set, there were 6 controls and 3 cases with the TT genotype, 14 controls and 16 cases with the CT genotype, and 10 controls and 11 cases with the CC genotype. Segregated by ancestry, the genotypes were: African American (CC: 10, CT: 16, TT: 3); Caucasian (CC: 4, CT: 5, TT: 2); Hispanic (CC: 5, CT: 8, TT: 1); and Haitian (CC: 2, CT: 1, TT: 3). For the TT genotype, two of the three CpGs associated with higher methylation levels in the cases compared to the controls were also statistically significant: CpG-3 (+12.6%, *P* = 0.04), CpG-4 (+12.0%, *P* = 0.04), and CpG-5 (+12.3%, *P* = 0.09) (Figures 3B–D). Methylation differences (<3.7%) between cases and controls in the CT and CC genotypes did not reach statistical significance. We also formally assessed whether the associations were statistically significant between the controls and cases (both were matched by maternal age, parity, ancestry, and socioeconomic status) by including an interaction term for the recessive TT genotype and case status. Due to our small sample size, we only assessed these interactions in the full sample of 60 individuals and observed significant interaction for all three CpGs: CpG-3 (*P* = 0.0009), CpG-4 (*P* = 0.0002), and CpG-5 (*P* = 0.0012).

To determine whether the observed methylation differences at intron 5 were due to maternal disorder-dependent changes in the cell-type composition of cord blood, pyrosequencing analysis of five candidate CpGs that were used previously to distinguish five blood cell types (T-cells, B-cells, NK cells, monocytes, and granulocytes) (Jaffe and Irizarry, 2014; Lee et al., 2018) was performed, and no differences in methylation between the cases and controls (*P* > 0.34, Supplementary Figure 1A) nor among the three genotypes were observed (*P* > 0.62, Supplementary Figure 1B).

Additionally, we sought to determine whether the mothers’ affective disorder diagnosis also affected the maternal epigenome by examining matched maternal blood samples controlled for maternal age, socioeconomic status, and ancestry. Interrogation of the maternal *FKBP5* intron 5 CpGs showed no significant differences with prominent hypomethylation in both cases and controls, as previously reported (Supplementary Figure 2; Klengel et al., 2013). Therefore, no additional analyses were performed in the maternal blood samples.

### Glucocorticoid-Induced Change in Intron 5 DNA Methylation and *FKBP5* Expression in an Embryonic Cell Line

**FIGURE 4:**
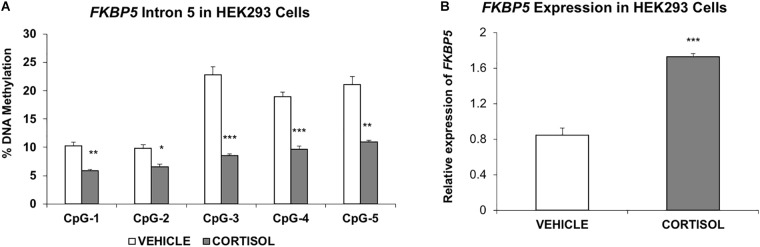
Studies in the human embryonic kidney 293 cell line **(A)**. Methylation of *FKBP5* intron 5 is decreased in cells treated with cortisol compared to vehicle solution **(B)**. RT-PCR results show increased expression of *FKBP5* corresponding to decreased methylation of intron 5. The bar graphs are represented as mean ± SEM. ^∗^indicates *P-*value < 0.05, ^∗∗^indicates *P-*value < 0.01, and ^∗∗∗^ indicates *P-*value < 0.001.

Last, to provide additional support for the susceptibility of intron 5 to glucocorticoid-induced changes in DNA methylation, a human embryonic kidney (HEK293) cell line was treated with the glucocorticoid cortisol for 7 days. Results showed significant decrease in methylation at all intron 5 CpGs examined: CpG-1 (4.5%, *P* = 0.003); CpG-2 (3.2%, *P* = 0.014); CpG-3 (14.3%, *P* = 0.0006); CpG-4 (9.3%, *P* = 0.0006); and CpG-5 (10.2%, *P* = 0.002, Figure 4A). Further, quantitative PCR showed a concomitant increase in expression of *FKBP5* (205% increase, *P* = 0.0006) (Figure 4B).

## Discussion

To explore a possible gene-by-environment relationship of “toxic stress” *in utero*, our work focused on the genotypic influence of rs1360780 on *FKBP5* methylation. In the full set of cord blood samples, we observed a significant recessive genotype effect with increased methylation at a subset of the five CpGs located in intron 5.

The mean methylation for the heterozygous and homozygous CT and CC genotypes were similar, which is consistent with prior work (Binder et al., 2004). However, the direction of association between the TT genotype and intronic methylation was positive in our cord blood samples. Previous work in the adult blood has shown that the T allele is correlated with decreased methylation and subsequently increased expression of *FKBP5*, conferring an increased risk for psychopathology (Klengel et al., 2013).

The rationale for focusing on the intronic regions of the gene is supported by the identification of glucocorticoid response elements (GREs) within introns (Polman et al., 2013), which may recruit currently unidentified transcription factors and lead to the looping of the intronic, GR-bound GREs to the promotor and each other to mediate glucocorticoid-induced transcription (Oakley and Cidlowski, 2013). Such glucocorticoid-mediated looping of the intronic GREs was demonstrated for *FKBP5*, and the strength of the association was allele-specific when the role of the SNP rs1360780 (C/T) located in the second intron of *FKBP5* was considered, indicating that this SNP moderates glucocorticoid-induced epigenetic changes at multiple intronic GREs, including GREs in intron 7 (Klengel et al., 2013) and in this case in intron 5. We emphasize that intron 5 harbors a highly conserved GRE of *FKBP5* that likely has a developmental role, which is suggested by the hypomethylation of this region in adults noted in our study and that of others (Klengel et al., 2013), compared to higher methylation levels in the cord blood DNA. In light of the demonstrated model, increase in DNA methylation at intron 5 would attenuate the binding of GR and/or other factors that mediate the promoter-intron interactions. Individuals with the TT genotype and increased methylation of intron 5 would be expected to show a blunted stress response following provocation of their HPA axis during times of stress. We reasoned that formal Bonferroni correction is not necessary, given the redundancy in measuring nearby correlated CpGs (Haerter et al., 2014), and each PCR amplicon was designed to interrogate a single region that consisted of several consecutive CpGs. Additionally, we measured methylation at the promoter, intron 1, intron 2, and intron 7 implicated in other studies. These additional CpGs that were measured represent 4 independent regions. A reasonable Bonferroni correction could be 0.05/5, corresponding to an alpha threshold of 0.01.

For the full set of samples, there was a significant association between the *FKBP5* intron 2 rs1360780 genotype and the methylation levels at three CpG sites located in intron 5. Our results suggest that rs1360780 functions in *cis* as a methylation quantitative trait locus (*cis*-meQTL), where the genotype (or alleles) influences the methylation levels of specific CpG sites over a distance of about 37.7 kilobases.

To understand the impact of adverse *in utero* environment on DNA methylation, analysis was subsetted to case status to gain insight to a potential gene-environment interaction during the key stages of development likely reflected in the cord blood samples. Cord blood samples of newborns whose mothers were diagnosed with affective disorders (cases) showed increased effect sizes for the TT genotype. This suggests that *in utero*, a specific genotype may be more sensitive to changes in the environment than during other developmental stages (Martin, 2000). It should be emphasized that when segregating data into case and control status, our sample size was small, especially with respect to the number of cases with the TT risk genotype.

Reduced methylation of intron 5 is noted in the brain and in mouse models; however, our work shows low baseline levels of DNA methylation with no significant differences between maternal cases with a diagnosis of affective disorders and healthy controls. It is not clear as to why the maternal blood samples lack epigenetic changes associated with affective disorder. One possibility is that the methylation changes are tissue-specific, and the blood is not reflective of epigenetic modifications at intron 5 of *FKBP5* in the brain. However, epigenetic differences were observed in the cord blood, and this region may lose methylation throughout development. Perhaps epigenetic modification at this region is critical for developing a normal physiologic response to stress. Our findings may suggest early life epigenetic plasticity to mediate an adaptive response. This illustrates the dynamic nature of methylation during periods of development that may become more fixed after a certain age.

While strictly speculative, the increased methylation of CpGs in the presence of the risk allele noted in the cord blood suggests the possibility of a compensatory response at key stages of accelerated development that initially is adaptive to the prenatal and neonatal environments and could later become maladaptive in childhood or adulthood. For example, in mice there appears to be a critical period in adolescence when methylation changes are more plastic (Niwa et al., 2016). This is well exemplified by the development of obesity in adults after exposure to malnutrition *in utero* during the Dutch Famine, in which key genes in metabolism and growth may initially be up- or downregulated in an adaptive fashion and then become maladaptive when food is plentiful (Ravelli et al., 1999; Heijmans et al., 2008). Results from our study raises the intriguing question of whether the methylation differences are associated with alterations in neurodevelopment and stress response in the infants. Studies of prenatal stress in animals show mixed behavioral outcomes in terms of resilience, with one showing resilience in females only (Zuena et al., 2008) and another showing males becoming predisposed but better adapted to subordinate status (Scott et al., 2017). Unfortunately, there is no information available on any stress-related outcomes, mental or physical health, or sex of the BBC infants. More longitudinal studies following methylation profiles in animal models and, more importantly, in humans may shed light on the critical periods during which methylation changes are more active and heavily influence the overall long-term fitness of the organism.

Human cell line data reproduced demethylation of *FKBP5* as was observed during childhood exposure to trauma. Although not derived from blood tissues, the cell line was embryonic in origin and capable of demonstrating glucocorticoid-induced loss of DNA methylation at the same intron 5 CpGs as in the cord blood. Additionally, the cells functionally showed an increase in expression in association with demethylation at intron 5. These findings also illustrate the importance of the overall context of human exposure to produce an effect on methylation including the underlying genetics, which exemplifies the connection between the epigenetic modifications and unmodifiable genetics of the individual. Genetics may also play a role in the degree of compensatory changes in methylation as suggested by our data through an undetermined process that occurs during fetal development.

In the case of *FKBP5*, studies show different genotypes have various levels of susceptibility to environmental stress and transcription of the gene, thereby contributing to hypercortisolemia and glucocorticoid resistance. Our data show that perhaps *in utero* and neonatally, genetics influences individual differences in susceptibility to environmental stress, and at times of increased plasticity, an adaptive response of the gene may predominate in those at increased genetic risk. However, the small sample size makes it difficult to comment on this possibility with certainty.

Our study has several limitations. As mentioned above, the small sample size, especially in the cases with the TT genotype, limited our ability to determine the role of the interaction between the genotype and the *in utero* environment on DNA methylation. Second, we only examined one SNP in the *FKBP5* gene. Rs1360780 was chosen based on several lines of evidence linking this SNP to psychiatric and glucocorticoid-related disorders. Additional *FKBP5* SNPs have been implicated in depression, PTSD, and exposure to childhood trauma (Binder et al., 2008; Zimmermann et al., 2011; Dackis et al., 2012). It is possible that other SNPs might exhibit similar gene-environment interactions on DNA methylation as was observed with rs1360780. Third, several case samples in the discovery set were obtained from preterm births. Although gestational age and birthweight were used as covariates in all of the analyses, it is possible that these two factors can still have a substantial effect on DNA methylation in the cord blood. Studies focused on the impact of preterm births on the epigenome are needed to elucidate the various epigenetic processes that occur during the different periods of gestation. Fourth, we did not perform additional assays to determine whether the observed methylation changes may be due to CpG hydroxymethylation (5-hmC). Without a prerequisite oxidation step (Booth et al., 2012), the current bisulfite conversion process cannot discriminate between methylation and hydroxymethylation. While 5-hmC is most abundant in the brain, it is possible that 5-hmC may play a role in the infant cord blood. In fact, a recent study found a negative association between 5-hmC content and aging in the human blood (Xiong et al., 2015), suggesting that cord blood samples may have higher levels of 5-hmC than adult blood. If true, this would undermine our conclusion that the increase in DNA methylation observed in the TT genotypes is adaptive. Oxidative bisulfite sequencing and supporting gene expression studies can provide a definitive answer. Finally, we acknowledge the technical limitations of conducting methylation analysis of single CpG probes for assessing changes in cell-type composition in the cord blood samples. Since all of our discovery set DNA samples had been exhausted by promoter and intronic methylation assays, we could only use the validation set for cell-type composition analysis. However, a genotype effect was not observed in the few TT genotypes found in the validation set. Although there were no significant differences in cell-type specific methylation levels, small sample size in the validation set, especially that of the TT genotype, hindered our ability to draw clear conclusions regarding the contribution of different cell types to the observed methylation levels. We also acknowledge the limitations of adapting cell-type methylation data, which have been characterized in adult blood, on cord blood samples. Several confounding factors, such as the presence of stem cells and nucleated red blood cells in the cord blood (Glasser et al., 2015), differences in the composition of cell types between adult and cord blood samples, and potential differences in baseline methylation levels at the five CpG probes between adult and cord blood samples all serve to weaken the effectiveness of using this deconvolution approach on cord blood samples.

In summary, we found that cord blood samples derived from mothers with affective disorders showed higher DNA methylation in a genotype-specific way. Future longitudinal studies correlating stress exposure and methylation changes *in utero* and during stages of childhood development have the potential to elucidate critical periods of epigenetic plasticity. Our data suggests that during the critical period of *in utero* development, the gene-environment association at *FKBP5* may be adaptive.

## Ethics Statement

This study was carried out in accordance with the recommendations of The Children’s Memorial Hospital Institutional Review Board (IRB), the BMC IRB, and the Massachusetts Department of Public Health, with written informed consent from all subjects. All subjects gave written informed consent in accordance with the Declaration of Helsinki. The protocol was approved by The Children’s Memorial Hospital Institutional Review Board (IRB), the BMC IRB, the Massachusetts Department of Public Health, and the Johns Hopkins School of Public Health IRB.

## Author Contributions

JD wrote the manuscript. JD, RL, and XW contributed to the conception and study design. OC performed the methylation and genotyping assays. YJ extracted relevant sample information from the Boston Birth Cohort. FS performed all of the statistical analyses.

## Conflict of Interest Statement

The authors declare that the research was conducted in the absence of any commercial or financial relationships that could be construed as a potential conflict of interest.

## Footnotes

1 https://www.r-project.org/

## Supplementary Material

Figure S1 Methylation levels at five candidate CpGs used to characterize five different cell types in the validation set cord blood. Each CpG representing a CpG probe on the Illumina 450K platform was chosen based on previous studies showing low methylation levels in one cell type and high methylation levels in the other four. Methylation levels for each CpG probe have been categorized by case status **(A)** and genotype **(B)**. The bar graphs are represented as mean ± SEM.

Figure S2 Methylation levels of *FKBP5* intron 5 CpGs in the maternal blood samples. Results show hypomethylation (<5%) of the intron 5 CpGs with no differences between cases and controls. The bar graphs are represented as mean ± SEM.

Table S1 Genomic coordinates (UCSC Genome Browser: GRCg37/hg19 Assembly) and primer sequences for FKBP5 pyrosequencing and genotyping on Chromosome 6.

Table S2 BBC cohort data.

Table S3 Pyrosequencing data for the discovery set.
