# Supplementary figures and images for: Effect of Genotype and Maternal Affective Disorder on Intronic Methylation of FK506 Binding Protein 5 in Cord Blood DNA
Source: Front Genet. 2018 Dec 17;9:648. doi: 10.3389/fgene.2018.00648 (PMC6305129; doi:10.3389/fgene.2018.00648)

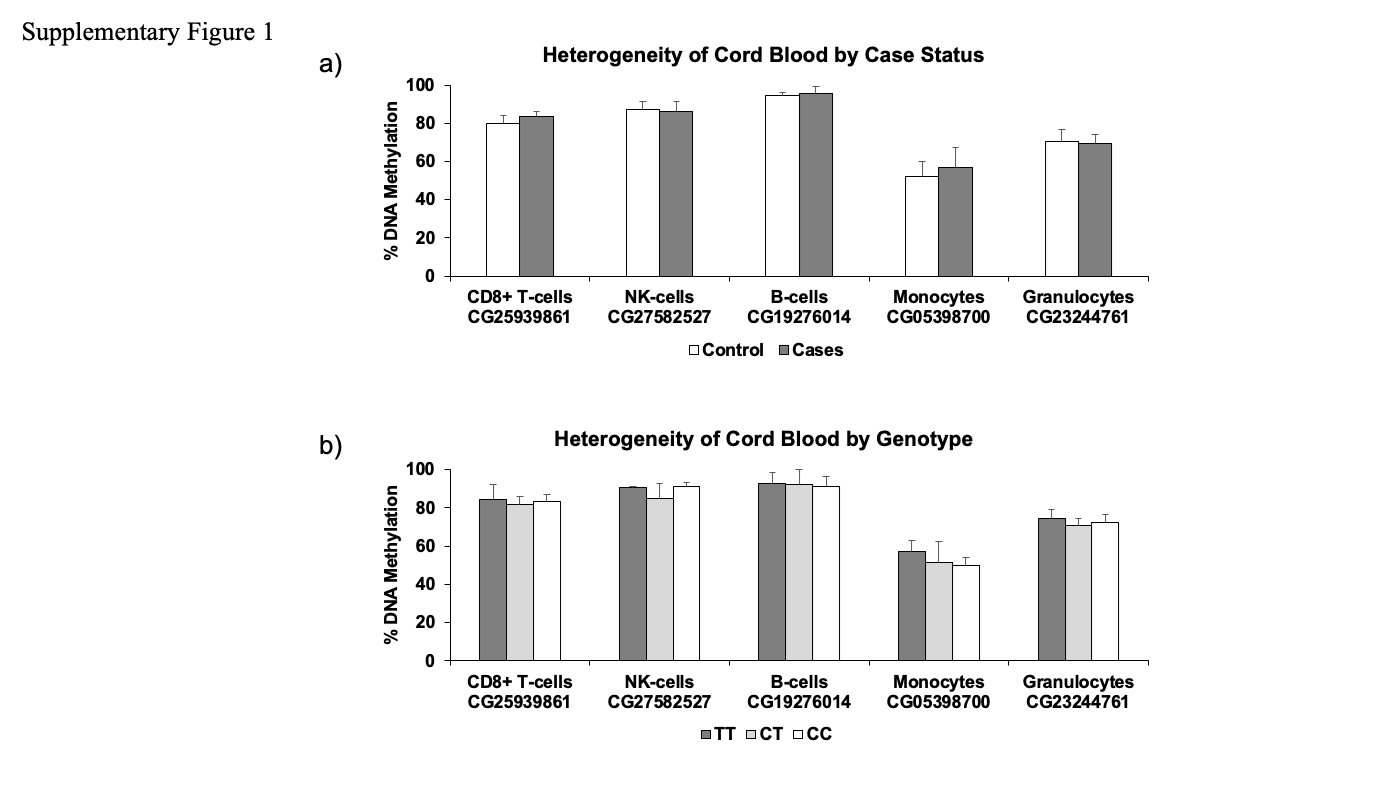

Supplement: Figure S1 — Methylation levels at five candidate CpGs used to characterize five different cell types in the validation set cord blood. Each CpG representing a CpG probe on the Illumina 450K platform was chosen based on previous studies showing low methylation levels in one cell type and high methylation levels in the other four. Methylation levels for each CpG probe have been categorized by case status (A) and genotype (B). The bar graphs are represented as mean ± SEM. [file Image_1.tiff]

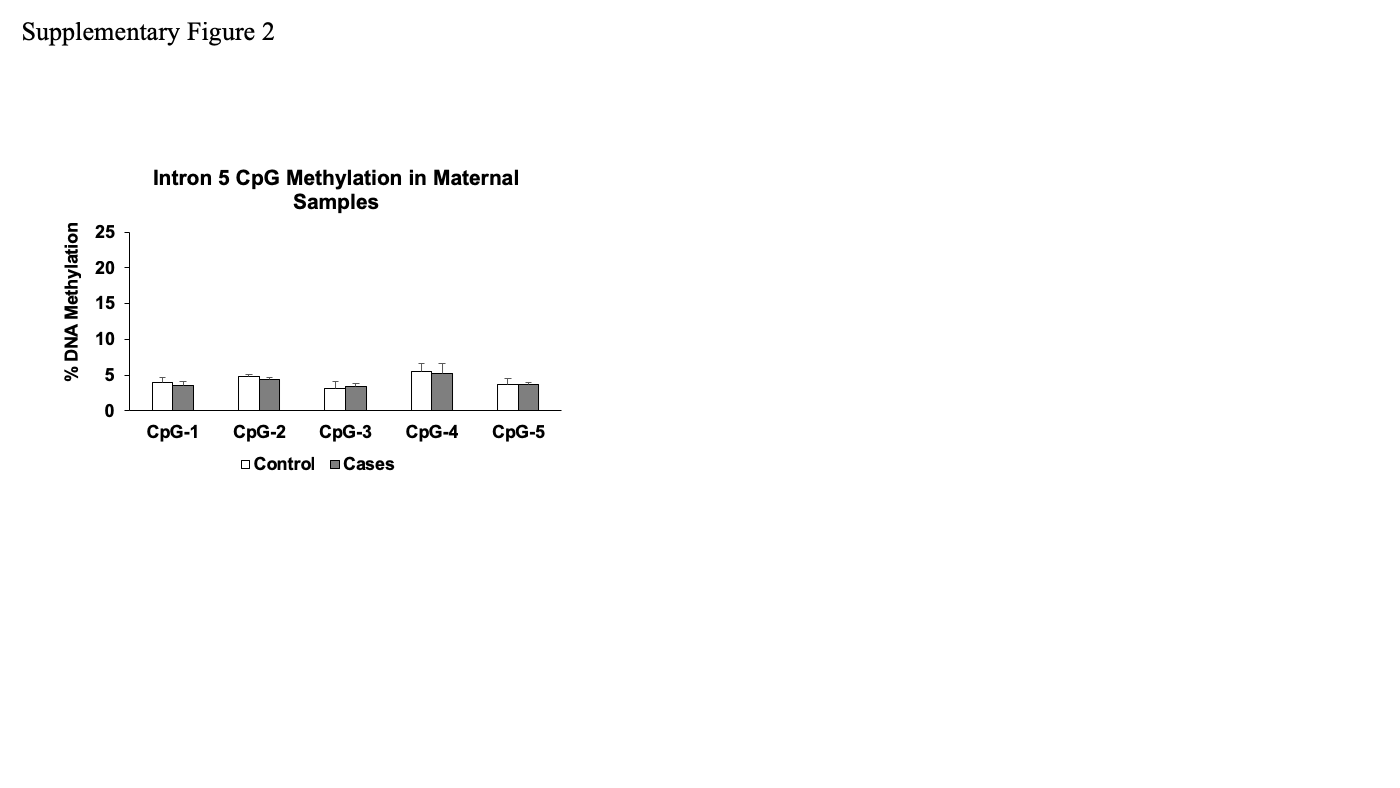

Supplement: Figure S2 — Methylation levels of FKBP5 intron 5 CpGs in the maternal blood samples. Results show hypomethylation (<5%) of the intron 5 CpGs with no differences between cases and controls. The bar graphs are represented as mean ± SEM. [file Image_2.tiff]
