# Supplementary material for: Effect of Genotype and Maternal Affective Disorder on Intronic Methylation of FK506 Binding Protein 5 in Cord Blood DNA
Source: Front Genet. 2018 Dec 17;9:648. doi: 10.3389/fgene.2018.00648 (PMC6305129; doi:10.3389/fgene.2018.00648)
Supplement: Table S1 — Genomic coordinates (UCSC Genome Browser: GRCg37/hg19 Assembly) and primer sequences for FKBP5 pyrosequencing and genotyping on Chromosome 6. [file Table_1.docx]

| **Location** | **PCR Primers** | **Pyrosequencing Primers** |
| --- | --- | --- |
| Promoter  35,657,180-35,657,570 | 5’-GTAGGTTAGGTTGTTTGTTAGGTTAGT-3’  5’-CCAAATACAACTCCTACATCTCTATA-3’  5’-TGGTTAGGTTAGTTTTAGGAAGTAAT-3’  5’-CAAAAATAACCATCTAATTAACAATCTACC-3’* | 5’- AGTAATTTTATTAAGTTTAAGATGT -3’  5’- GGAGATTAAAATAGGAAGAGGTTTTA -3’  5’- GTTTGTAATTTTAGTATTTTGGAAGG -3’  5’- TAGTTAATATGGTGAAATT -3’  5’- AAATATAAAAATTAGTTGGGTTTGGTG -3’  5’- GGGAGGTTGAGGTAGGAGAA -3’  5’- TGGGAGGTAGAGGTTGTAGTGAG -3’ |
| Intron 1  35,655,608-35,655,700 | 5’-AGTTTAAATTGTTTTATGTAGAATTTATTGA-3’  5’-TCACTCCCAAACCATACC-3’  5’-GTTTTGAATTATATTGAAGGGTATTT-3’  5’-CAAAACTCCTTATACTCTTCTATTCTAA-3’* | 5’- ATAATTTGAAATTTATTA -3’  5’-GTAGAATTYGATTTTAGAGA-3’  5’-GAAGAAAGTTATTTTGTATTTTG-3’ |
| Intron 2 Region 1  35,605,303-35,605,534 | 5’-GGTTTAATGTTTTATATTTTGATAGTA-3’  5’-CTTTACTTAAACTTCTTTTTAAATA-3’  5’-GTTTTGTAGTTTAGGTTGGAGTGTAATG-3’  5’-AAAATCCACCATTCTTTCTAATCT-3’* | 5’-GGTTGGAGTGTAATGGTA-3’  5’-GAGTAGTTGGGATTATAGG-3’  5’-ATTATGTTGGTTAGGTTGGTT-3’  5’-GATTATAAGTATGAGTTA-3’ |
| Intron 2 Region 2  35,606,441-35,606,662 | 5’-GAGTAGTAGAGATTATAGGTATATGT-3’  5’-ATAACTCTCTAACTCTCTAATAACC-3’  5’-GTTGGTTTGGAATTTTTGAGTTTTAATG-3’  5’-ATTCATCCCACATACCCAATTACTA-3’* | 5’-TGGAATTTTTGAGTTTTAATGA-3’  5’-TGAGATTATTGGTATAAGTT-3’  5’-GATATTATATAATATTTTATAAGTGT-3’  5’-GAGGTTATGTTTAATTGTAAGTT-3’ |
| Intron 2 Region 3  35,607,855-35,608,021 | 5’-GTGTTAATAATGGTGTAGGAGAAATAAT-3’  5’-CCTTTTATTACTATACCTTTTTCTATA-3’  5’-TAAGATGGAAGAATTAAGAGATGT-3’  5’-TTACCTACAATATTCAATACAATAACAC-3’* | 5’-AGAAATAAAATAAAAGAAATGT-3’  5’-GAAATATATATAGTTATGTA-3’  5’-ATAGGGATATGTTTTGAGAAATGTG-3’  5’-GTAGAGTGTATTTATATAAATTTAGATGG-3’ |
| Intron 2 Region 4  35,609,503-  35,609,664 | 5’-AAGTAAGATATATTTTAAGAGGAA-3’  5’-TATAATCTCAACTCACTACAACCT-3’  5’-AAGAGGAAATAGTAAAAGATATTTTTATTG-3’*  5’-CCTCCTAAATTCAAACAATTCTACC-3’ | 5’-TCTACCTCAACCTCC-3’  5’-ACTCCTAACCTCAAATAATC-3’  5’-TTACAAACATAAACCAC-3’ |
| Intron 5  35,569,751-  35,569922 | 5’-GGTAGAGAAAGAAATAAATAAGTTA-3’  5’-TTCTTACATTTCATTTTTATTACTACTA-3’  5’-AAGATTATGTAATTTAAAGGGGGAGGG-3’*  5’-CTCTCTTTCCTTTTTTCCCCCCTAT-3’ | 5’-TCTTTCCTTTTTTCCCCCCTATT-3’  5’-CAATTTAAATAATATTTTACAACT-3’ |
| Intron 7  35,558,386-  35,558,721 | 5’-AGAGTGAAATTGAGATGGAAATATGT-3’  5’-AATTTCTTCTCCATCCACTTCCTATA-3’  5’-AGGAGGTATGTTGTTTTTGGAATTTAAG-3’  5’-AATTTATCTCTTACCTCCAACACT-3’* | 5’-GGAGAAGTATAAAAAAAAAATGG-3’  5’-GTTATAGAGTTTAGTGGTTT-3’  5’-GGAGTTATAGTGTAGGTTTT-3’  5’-TTAAGGAGTTATTTGGTAGA-3’  5’-TGATATATAGGAATAAAATAAGAAT-3’ |
| rs1360780  35,607,571 | 5’-CAATTCACATATATTCAGAAGAGATC-3’  5’-CAGCAGTAGCAAGTAAGAATT-3’* | 5’-AAGGCTTTCACATAAGCAAAGTTA-3’ |

**Supplementary Table 1.** Genomic coordinates (UCSC Genome Browser: GRCg37/hg19 Assembly) and primer sequences for FKBP5 pyrosequencing and genotyping on Chromosome 6.

*Biotinylated and HPLC-purified primer.
